# Mechanism of Peppermint Extract-Induced Delay of ‘Packham’s Triumph’ Pear (*Pyrus communis* L.) Postharvest Ripening

**DOI:** 10.3390/foods13050657

**Published:** 2024-02-21

**Authors:** Chenglin Liang, Fudong Jiang, Hongpeng Xu, Zan Zhang, Wei Tian, Haifeng Sun, Yali Jing, Mengzhen Wang, Yingyu Zhuang, Dingli Li, Jianlong Liu

**Affiliations:** 1 Haidu College, Qingdao Agricultural University, Laiyang 265200, China; ljl2015lcl@163.com (C.L.); zhangzan0919@163.com (Z.Z.); tw17616226889@163.com (W.T.); sunhaifeng0629@163.com (H.S.); 19853547195@163.com (Y.J.); 19853535820@163.com (M.W.); mary020230@163.com (Y.Z.); 2 Yantai Academy of Agricultural Sciences, Yantai 265500, China; jiangfudong0505@163.com; 3 College of Horticulture, Qingdao Agricultural University, Qingdao 266109, China; 20212102044@stu.qau.edu.cn (H.X.); lidingli@qau.edu.cn (D.L.)

**Keywords:** pear, *Mentha haplocalyx*, fruit ripening, metabolome, transcriptome

## Abstract

Postharvest ripening is correlated to the quality and shelf life of European pear fruit. In this study, the effects of peppermint extract on fruit phenotype, related physiological activities, and aroma components during postharvest ripening of the European pear variety ‘Packham’s Triumph’ were examined. Fruit treated with 2.0 g L^−1^ peppermint extract for 12 h showed delayed softening by 4 d compared with that of the untreated control group. The peak values of ethylene and respiratory rate in fruit were reduced to a certain extent after peppermint extract treatment; however, the peppermint extract did not delay the occurrence of the respiratory climacteric peak. Peppermint extract treatment also did not significantly increase the content of the characteristic peppermint aroma in pear fruit. Further, widely targeted metabolome analysis revealed 298 significantly different metabolites, with flavonoids (40%) and lipid compounds (15%) accounting for the highest proportion on the first day after treatment. The Kyoto Encyclopedia of Genes and Genomes pathway result showed significant enrichment in the metabolic pathways of biosynthesis of flavonoid, isoflavonoid, flavone and flavonol, linoleic acid, and alpha-linolenic acid metabolism following peppermint extract treatment. The combined analysis of transcriptome and metabolome data showed significant enrichment in linoleic acid metabolism and alpha-linolenic acid metabolism on the first, third, and fifth days after peppermint extract treatment. This study indicates that peppermint extract mainly affects the pear fruit softening process in the early stage after treatment.

## 1. Introduction

Pear is an important fruit tree in China [1]. With the improvement of living standards, the production focus of the pear fruit industry has gradually shifted from yield to quality. The European pear (*Pyrus communis* L.) belongs to the subfamily Maloideae of the family Rosaceae, which originated in Europe, the Mediterranean region, and Central Asia and is now widely cultivated globally, except in East Asia. ‘Packham’s Triumph’ is a type of European pear native to Australia, which is characterized by a soft and delicate flesh with few stone cells, along with aromatic and juicy properties. However, the fruit is hard in texture at harvest and needs to be completely matured and softened before it can be eaten. In actual production, once the fruit is completely softened, it will quickly rot and spoil, affecting its commercial value. Therefore, it is crucial to extend the time for fruit to reach the optimal hardness range.

The peppermint plant *Mentha haplocalyx* Briq. in the family Lamiaceae is widely distributed in the temperate regions of the Northern Hemisphere. Its dry above-ground parts are often used in traditional Chinese medicine as a surface relieving medicine. Mint has a wide range of uses, not only in the pharmaceutical field but also in the food, cosmetics, spices, tobacco, and other industries [2,3,4]. Its extract contains a large amount of antioxidant components, flavonoids, and polyphenols [5]. Numerous studies have reported that peppermint significantly protects nerve and cell vitality, limits protein and DNA damage, reduces lipid peroxidation, and maintains the activity of glutathione and superoxide dismutase (SOD) in the acute stage of oxidative stress induction [6,7,8]. In addition to its beneficial effects on the human body, mint extract is often used as a natural plant extract to replace traditional fungicides in the postharvest preservation process of fruit and vegetables. For instance, *Mentha spicata* and *Mentha piperita* were reported to exhibit strong inhibition on the growth of the creeping stem rot mould *Rhizopus stolonifer* by targeting its cytoplasmic membrane, reducing the rot of strawberry and peach root mould infection [9]. Mint essential oils with different concentrations (0.3, 0.6, 1.25, 2.5, and 5 μL mL^−1^) had an effective inhibitory effect on postharvest mango anthracnose infection. The severity of mango anthracnose on fruit treated with peppermint coating was similar to or even lower than that of fruit treated with the synthetic fungicides methoxybuzin (10 μg a.i. mL^−1^) and phenecycline (0.5 μg a.i. mL^−1^) [10]. Other studies have shown that mint exhibited good inhibitory effects on postharvest apricot brown rot, *Botrytis cinerea* infection of table grapes, and postharvest mould infection of cherry tomato fruit [11,12,13]. In addition to inhibiting postharvest fungal infection, mint extract also appears to have a significant effect on the preservation of postharvest fruit. Mint extract was found to maintain the quality of cherry tomato fruit [11]. In addition, the soluble protein, catalase (CAT), and peroxidase (POD) activities in fresh cut carnation flowers treated with mint leaf extract significantly increased, thus improving the lifespan of the flowers [14]. Compared with those of untreated papaya, peppermint-treated papaya showed reduced hardness and weight loss, maintained total soluble solids and enzyme activity, and delayed changes in flesh and skin colour during storage; notably, the mint extract did not affect the sensory quality of the papaya [15]. Similarly, during the storage process of sweet cherries, mint extract treatment was found to reduce the weight and hardness loss of the fruit, while improving the overall antioxidant activity, with no negative impact detected on the content of bioactive compounds and the sensory characteristics of the fruit [16]. The above studies indicated that peppermint had great potential for application in the postharvest process. These beneficial effects are considered to be closely related to the fact that peppermint extract is enriched in various antioxidant substances, which could enhance the antioxidant capacity of fruit.

Fruit softening is a complex process that takes place during ripening [17]. In addition to softening, fruit undergoes changes to colour, flavour, and aroma during this stage. Therefore, regulation of the softening process has very important implications for fruit texture, shelf life, and consumer acceptance [18]. Numerous enzymes and proteins associated with cell wall loosening have been identified in fruit, including pectin methylesterase (PE), polygalacturonase (PG), β-galactosidase (β-GAL), α-l-arabinofuranosidase (α-L-Af), cellulase (Cel), expansin, and xyloglucan endotransglycosylase [19]. Fruit softening is considered to be the result of synergistic actions among these enzymes and proteins [20]. In addition, fruit softening has been considered an important manifestation of fruit senescence. During this process, pulp cells accumulate a large amount of reactive oxygen species, which disrupt the normal physiological metabolism of the fruit, leading to fruit decay and spoilage [21,22,23]. To date, various methods have been investigated for optimising fruit and vegetable preservation, including efforts to delay the postharvest ripening process of fruit by inhibiting ethylene and respiration or by applying exogenous antioxidants. For example, 1-methylcyclopropene is commonly applied to pears to delay fruit ripening by inhibiting the ethylene signalling pathway [24,25]. In addition, the use of plant growth regulators such as salicylic acid and melatonin has been shown to extend the shelf life of fruit by enhancing their antioxidant capacity [26,27,28]. However, since these substances are mostly chemical agents, their extraction is difficult, and their broad application is limited by high costs. By contrast, mint extract is a natural plant component, which has been widely established to not affect the sensory quality of fruit, making it a green and safe alternative as a biological agent. However, mint extract has not yet been applied in the postharvest softening process of pears, especially European pears, and the specific mechanism of action is also unclear.

Therefore, the aim of this study was to investigate the effects of peppermint extract on fruit through phenotype observations and changes in fruit physiological indicators. Further, by using a combination of metabolomics and transcriptomics analysis, the impact of peppermint extract on the metabolic changes of the European pear ‘Packham’s Triumph’ was comprehensively analysed. Combined with transcriptional regulation, the pathway of action of peppermint extract was systematically revealed, laying a theoretical foundation for its application in the postharvest storage of pear.

## 2. Materials and Methods

### 2.1. Plant Materials and Treatments

Mature fruit of ‘Packham’s Triumph’ (*Pyrus communis* L.) was collected as the study material from a pear orchard in Yantai, Shandong province, China (E 121°37′58.08″, N 37°17′8.95″). ‘Packham’s Triumph’ fruit was harvested on 10 October at the stage of its commercial harvest maturity. The harvested fruit was randomly packed in boxes and then immediately treated by immersion in a 2.0 g L^−1^ mint (*Mentha haplocalyx*, MH) solution or distilled water as the negative control (CK) at 25 °C for 12 h. Following treatment, the fruit was stored and monitored throughout its shelf life at 25 °C.

### 2.2. Preparation of MH Solution

Dried mint was weighed to 20 g, added to 0.3 L distilled water, and then soaked for 1 h. After heating to boiling, boiling was continued for 30 min with a slow fire and filtered, and the filtrate was retained. The filter residue was mixed with 0.1 L distilled water, heated to boiling, and then cooked for 30 min over a gentle fire. These two filtrates were combined and concentrated to 0.1 L under reduced pressure to obtain the original solution, which was stored at 4 °C for further experiments.

### 2.3. Morphological Observations

The morphological differences between CK fruit and MH-treated fruit were observed using flesh cross-cut sections. Photographs of the fruit were taken at 1, 3, 5, and 7 d after treatment.

### 2.4. Ethylene Production, Respiration Rate, and Flesh Firmness Measurements

Ethylene production and respiration rates of the fruit were measured every day throughout storage at 25 °C up to 11 d after treatment as described by Xie et al. (2015) [29]. Ten fruit from each group served as one replicate, and five replicates were adopted at each measuring point.

The firmness, related enzyme activities, and related gene expression levels were detected at 1, 3, 5, 7, 9, and 11 d after treatment. Flesh firmness was detected by a GY-3 fruit hardness tester (Quzhou Aipu Measuring Instrument Co., Ltd., Quzhou, China), averaged from five fruit in each replicate with five replicates adopted.

### 2.5. Antioxidant Enzymes and Cell Wall Metabolism Enzyme Assays

For total RNA extraction and the antioxidant enzyme assay, flesh samples were collected from three fruit for each replicate and subsequently frozen in liquid nitrogen and stored at −80 °C; three biological replicates were adopted. 

The activities of SOD (EC 1.15.1.1), POD (EC 1.11.1.7), CAT (EC 1.11.1.6), ascorbate peroxidase (APX; EC 1.11.1.11), pectinesterase (PE; EC 3.1.1.11), β-GAL (EC 3.2.1.23), and α-L-Af (EC 3.2.1.55) were assayed using corresponding enzyme activity assay kits (Beijing Solarbio Science & Technology Co., Ltd., Beijing, China) according to the manufacturer’s instructions.

### 2.6. Real-Time Quantitative Polymerase Chain Reaction (RT-qPCR)

The total RNA of the pear flesh was extracted using RNAprep Pure Plant Kit (Tiangen Biotech Co., Ltd., Beijing, China) according to the manufacturer instructions. The HiScript II 1st Strand cDNA Synthesis Kit (Vazyme Biotech Co., Ltd., Nanjing, China) was used to synthesise single-stranded cDNA. The Lightcycler^®^ 480 II System (Roche, Basel, Switzerland) and the ChamQ SYBR Color qPCR Master Kit (Vazyme Biotech Co., Ltd., Nanjing, China) were then used to detect relative gene expression levels. The reaction protocol was as follows: 95 °C for 5 min, followed by 45 cycles at 95 °C for 15 s, 60 °C for 30 s, and 72 °C for 30 s. *ACTIN* was used as an internal reference. All RT-qPCR experiments were performed in triplicate using three biological replicates and three technical replications. Statistical significance was determined using Student’s *t*-test by IBM SPSS Statistics v21.0. The used primers are listed in Table S1.

### 2.7. Volatile Organic Chemical (VOC) Analysis by Gas Chromatography–Mass Spectrometry (GC-MS)

Flesh samples of pears in the CK and MH groups at 5 d after treatment were ground to a powder in liquid nitrogen and then used for pre-treatment. After sampling, desorption of the VOCs from the fibre coating was carried out in the injection port of the GC apparatus (Model 8890, Agilent, Santa Clara, CA, USA) at 250 °C for 5 min in splitless mode. The identification and quantification of VOCs was carried out using an Agilent Model 8890 GC and a 7000D mass spectrometer (Agilent), equipped with a 30 m × 0.25 mm × 0.25 μm DB-5MS (5% phenyl-polymethylsiloxane) capillary column. Helium was used as the carrier gas at a linear velocity of 1.2 mL min^−1^. The injector temperature was kept at 250 °C, and the detector temperature was maintained at 280 °C. The oven temperature was programmed from 40 °C (3.5 min), increasing at 10 °C min^−1^ to 100 °C, at 7 °C min^−1^ to 180 °C, and at 25 °C min^−1^ to 280 °C, and held for 5 min. Mass spectra were recorded in electron impact ionisation mode at 70 eV. The quadrupole mass detector, ion source, and transfer line temperatures were set at 150 °C, 230 °C, and 280 °C, respectively. Selected ion monitoring mode was used for the MS-based identification and quantification of analytes.

### 2.8. Metabolomic Analysis

The samples of pears in the CK and MH groups at 1, 3, 5, and 7 d after treatment were prepared using vacuum freeze-drying technology, and the sample extracts were analysed using an ultrahigh-performance liquid chromatography (UPLC)-electrospray ionisation (ESI)-tandem mass spectrometry (MS/MS) system (UPLC, ExionLC™ AD; MS, Applied Biosystems 6500 Q TRAP, Applied Biosystems, Inc., Carlsbad, CA, USA). The analytical conditions were as follows: UPLC column, Agilent SB-C18 (1.8 µm, 2.1 mm × 100 mm); the mobile phase comprised pure water with 0.1% formic acid (solvent A) and acetonitrile with 0.1% formic acid (solvent B). Sample measurements were performed with a gradient program that employed the starting conditions of 95% A. Within 9 min, a linear gradient to 5% A was programmed, which was maintained for 1 min. Subsequently, a composition of 95% A was adjusted within 1.1 min and maintained for 2.9 min. The flow velocity was set to 0.35 mL min^−1^. The column oven was set to 40 °C, and the injection volume was 2 μL. The effluent was alternatively connected to an ESI-triple quadrupole-linear ion trap mass spectrometer.

The ESI source operation parameters were as follows: source temperature, 500 °C; ion spray voltage, 5500 V (positive ion mode)/–4500 V (negative ion mode); ion source gas I, gas II, and curtain gas of 50, 60, and 25 psi, respectively; and high collision-activated dissociation. QQQ scans were acquired in multiple reaction monitoring (MRM) mode with the collision gas (nitrogen) set to medium. The declustering potential and collision energy for individual MRM transitions were further optimized. A specific set of MRM transitions were monitored for each period according to the metabolites eluted within this period.

The differential metabolites were determined according to a variable importance in projection value > 1 and absolute log2 fold change ≥ 1.0. Identified metabolites were annotated using the Kyoto Encyclopedia of Genes and Genomes (KEGG) Compound database (http://www.kegg.jp/kegg/compound/, accessed on 11 August 2023). Annotated metabolites were then mapped to the KEGG Pathway database (http://www.kegg.jp/kegg/pathway.html, accessed on 11 August 2023). Pathways with significantly regulated metabolites mapped were then subjected to metabolite set enrichment analysis, and their significance was determined by the *p*-value of the hypergeometric test. 

### 2.9. Transcriptomic Analysis

A plant total RNA isolation kit (TaKaRa, Beijing, China) was used to extract total RNA from samples, following the manufacturer’s instructions, and different samples were subjected to three biological replicates. After qualification using a bioanalyser (2100, Agilent, Santa Clara, CA, USA), 1 mg of each sample was used for cDNA library construction. Four RNA sequencing libraries were constructed for the dormant shoot tips (MP1 and OP1) and the buds in the expansion period (MP2 and OP2). For library construction, 1 mg of RNA per sample was used as the input material. Library quality was assessed with the Agilent 2100 Bioanalyzer system. The clean dataset was obtained by removing reads containing adapter sequences, poly-N, and low-quality reads from the raw data. The Q20, Q30, and GC contents of the clean data were calculated. All downstream analyses were based on the high-quality clean data. The reference genome (Pyrus communis Bartlett DH Genome v2.0) and gene model annotation files were downloaded from the GDR database (https://www.rosaceae.org/species/pyrus/pyrus_communis/genome_v2.0, accessed on 11 August 2023). An index of the reference genome was generated using HISAT2 v2.0.5 software, and paired-end clean reads were aligned to the reference genome using HISAT2 v2.0.5. FeatureCounts v1.5.0-p3 was used to count the read numbers mapped to each gene. The fragments per kilobase of exon per million mapped fragments value of each gene was calculated based on the length of the gene and the number of reads mapped to the gene. Differential expression analysis of two conditions/groups (two biological replicates per condition) was performed using the DESeq2 R package (v1.20.0). Gene Ontology (GO) enrichment analysis of differentially expressed genes (DEGs) was implemented with the clusterProfiler R package (v1.20.0) for which the gene length bias was corrected. Statistical enrichment of DEGs in KEGG pathways was detected with the clusterProfiler R package.

### 2.10. Statistical Analysis

The average values and standard deviations were calculated using Microsoft Excel 2016, and the data are presented as means ± standard deviation of at least three independent biological replicates. Statistical significance was determined using Student’s *t*-test by IBM SPSS Statistics v23.0.

## 3. Results

### 3.1. Effects of MH on Physiological Indicators of Postharvest Pear Fruit

**Figure 1:**
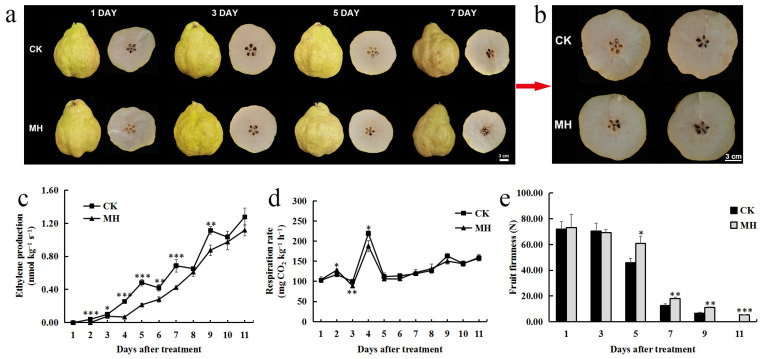
Phenotypes and physiological changes of postharvest ‘Packham’s Triumph’ pear fruit in the untreated control (CK) and 2.0 g L^−1^ *Mentha haplocalyx* extract (MH) treatment groups. (**a**) Phenotypes of fruit during storage. (**b**) Cross-section comparison of fruit on the eleventh day after treatment. (**c**) Ethylene content. (**d**) Respiratory rate. (**e**) Fruit firmness. * *p* < 0.05, ** *p* < 0.01, *** *p* < 0.001.

The peppermint extract delayed the softening process of the ‘Packham’s Triumph’ fruit. The fruit in the CK group showed waterlogging on the seventh day (Figure 1a), while the treatment group began to show waterlogging only on the 11th day (Figure 1b), representing a delay in fruit softening by 4 d compared with that of the CK group. Under room temperature storage conditions (25 °C), the ethylene release of the CK group showed a stepwise increase, while that of the treatment group was significantly reduced on days 2–7 (Figure 1c). Both the treatment and control groups had a respiratory climacteric peak on the fourth day of storage. The MH treatment did not result in a delay in the emergence of the respiratory climacteric peak; however, it did cause a decrease in the respiratory intensity (Figure 1d). The fruit hardness of the treatment group was significantly higher than that of the control group starting from day 5; on the eleventh day, the fruit hardness of the control group was close to 0, while the fruit in the treatment group still had a certain degree of hardness (Figure 1e).

### 3.2. Changes in Antioxidant Enzymes and Enzymes Related to Cell Wall Degradation

**Figure 2:**
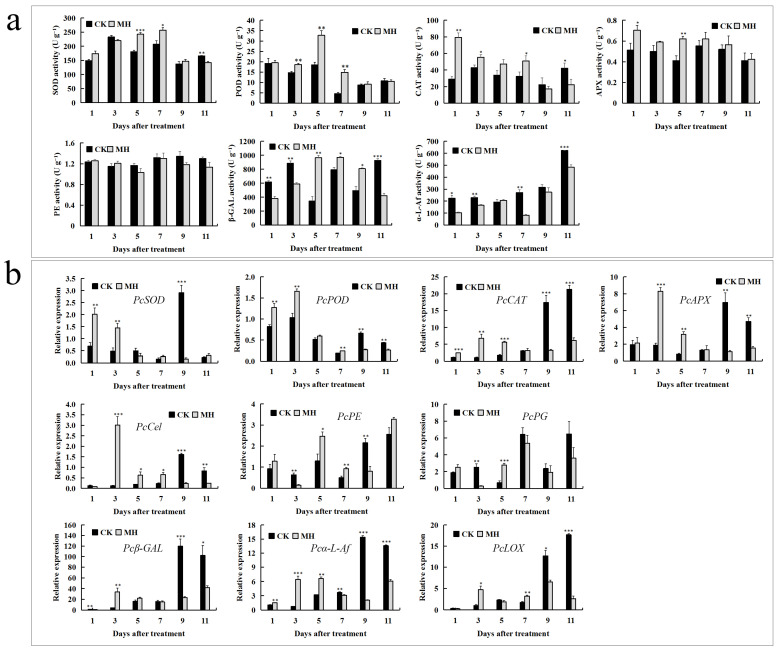
The effect of peppermint extract (MH) on antioxidant enzymes and enzymes related to cell wall degradation in ‘Packham’s Triumph’ pear fruit. (**a**) Activities of antioxidant enzymes and enzymes related to cell wall degradation. (**b**) Relative gene expression of antioxidant enzymes and cell wall degradation-related enzymes. * *p* < 0.05, ** *p* < 0.01, *** *p* < 0.001.

After soaking in mint extract, the SOD activity in the fruit was significantly higher than that in the control group on the fifth day, and the POD activity was significantly higher than that in the control group at 3, 5, and 7 d of storage. The CAT and APX activities were significantly higher in the MH group than those in the control group on the first day after treatment (Figure 2a). After treatment with MH extract, there was no significant difference in the activity of the PE enzyme related to cell wall degradation in the fruit; however, β-GAL activity was significantly lower than that of the control group on the first and third days after treatment, which then began to increase on the fifth day. The changes of α-L-Af activity were the most significant among the tested enzymes, with significant reductions compared with those of the control group detected on the first, third, seventh, and eleventh days after treatment (Figure 2a). Gene expression analysis showed that PcSOD and PcPOD levels were significantly increased in the first 3 d after peppermint extract treatment, and the expression level of PcCAT was significantly higher than that of the control group over the first 5 d of storage. The expression of PcAPX was significantly upregulated on the third and fifth days after treatment. The expression levels of the cell wall degradation enzyme-related genes PcCel, Pcβ-GAL, Pcα-L-Af, and PcLOX were significantly decreased in the treatment group on the ninth and eleventh days compared with those of the control group, and PcPE and PcPG expression showed significant downregulation on the third day of storage (Figure 2b).

### 3.3. Effects of MH on the Aroma Components of Postharvest Pear Fruit

**Figure 3:**
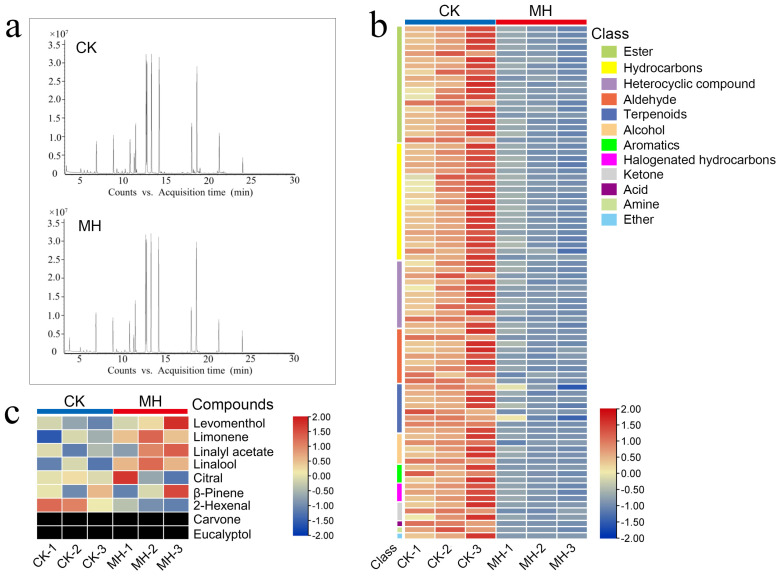
The effect of peppermint extract (MH) treatment on the aroma of pear fruit. (**a**) Ion peak diagram for aroma detection of pear fruit after treatment with peppermint extract. (**b**) Relative content heat maps of main aroma components in fruit. (**c**) Comparison of the relative content of characteristic mint volatile substances in pear fruit of the two groups. CK-1, CK-2, and CK-3 represent three repeats in the control group; MH-1, MH-2, and MH-3 represent three repeats in the treatment group.

To investigate whether peppermint extract has an effect on the aroma components of pear fruit, the aroma components were measured on the fifth day after treatment. The aroma content of the fruit treated with peppermint extract was significantly lower than that of the control group (Figure 3a,b), indicating that the fruit maturity of the treatment group was at a much lower stage than that of the control group at this time. Nine characteristic aroma components of peppermint were selected for comparison, demonstrating that the peppermint extract treatment did not significantly increase the content of characteristic peppermint aroma components in the pear fruit. The characteristic peppermint aroma components carvone and eucalyptol were not detected in the pear fruit (Figure 3c).

### 3.4. Metabolomic and Transcriptomic Analysis of Control and MH-Treated Pear Fruit

**Figure 4:**
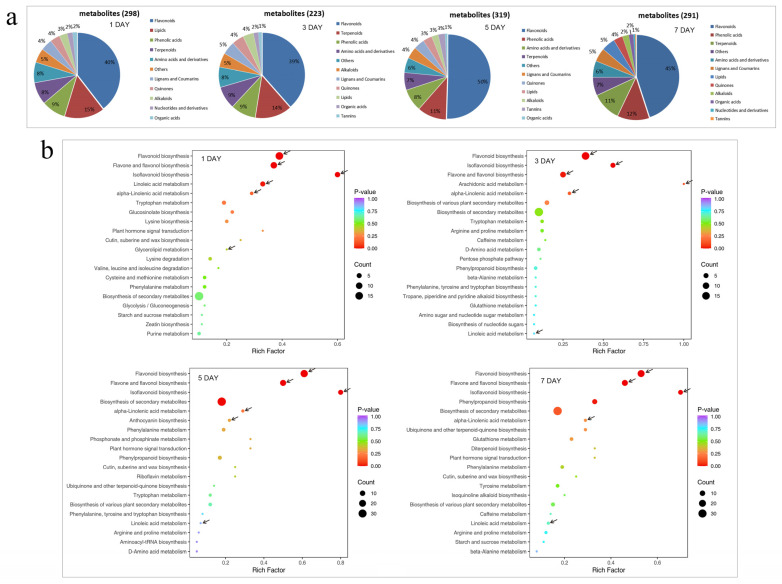
Metabolic changes in pear fruit after treatment with peppermint extract (MH). (**a**) Changes in differential metabolic components within fruit after treatment for different days. (**b**) Kyoto Encyclopedia of Genes and Genomes (KEGG) analysis of differential metabolites in fruit for different days of storage after treatment. Arrows represent significant enrichment pathways associated with softening.

The metabolic changes in pear fruit were analysed by metabolomics after peppermint extract treatment, showing differential changes in metabolic components in the treated fruit. On the first day after treatment, there were 298 significantly different metabolites, with flavonoids (40%) and lipid compounds (15%) accounting for the highest proportion. On the following third, fifth, and seventh days, there were 223, 319, and 291 differentially expressed metabolites, with flavonoids accounting for 39%, 50%, and 45% of the total, respectively (Figure 4a). KEGG enrichment analysis of differential metabolites showed that the metabolic pathways of flavonoid biosynthesis, flavone and flavonol biosynthesis, isoflavonoid biosynthesis, linoleic acid metabolism, and alpha-linolenic acid metabolism were significantly enriched on the first, third, fifth, and seventh days after treatment (Figure 4b).

**Figure 5:**
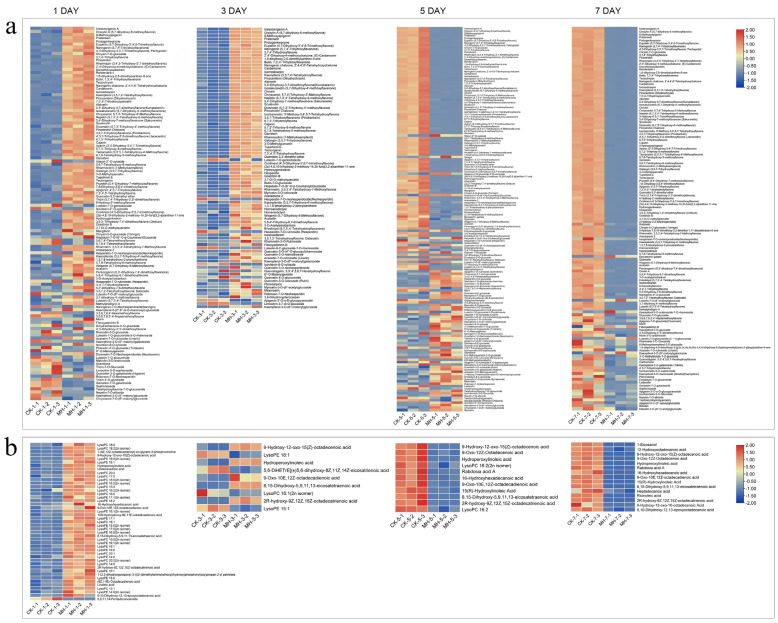
The effect of peppermint extract (MH) on the metabolism of flavonoids and lipids in fruit. (**a**) Changes in differential metabolites of flavonoids in pear fruit after treatment for different days. (**b**) Changes in differential metabolites of lipids in pear fruit after treatment for different days. CK-1, CK-2, and CK-3 represent three repeats in the control group; MH-1, MH-2, and MH-3 represent three repeats in the treatment group.

After treatment with peppermint extract, the flavonoid metabolites in the ‘Packham’s Triumph’ pear fruit significantly increased. The heat map in Figure 5a showed that the relative content of flavonoid substances in the fruit was significantly higher than that in the control group on the first and third days after peppermint extract treatment. However, on the fifth and seventh days, the flavonoid substances in the fruit of the control group significantly increased and were higher than those in the treatment group. Flavonoids such as naringin, kaempferol, pratensein, and diosmetin significantly increased on the first and third days but then decreased significantly on the fifth and seventh days (Figure 5a). The components related to lipid metabolism showed a similar change trend. On the first day after treatment with peppermint extract, the relative content of lipid metabolites in the fruit was significantly higher than that in the control group. On the third day, the difference in lipid metabolism decreased. On the fifth and seventh days, lipid metabolites accumulated in the control group, becoming significantly higher than those in the peppermint extract treatment group (Figure 5b). Among them, the levels of lysoPC (18:0, 18:2, 16:0, 20:0, 17:0, 15:0, 16:2, 16:1; 20:1, 14:0, 15:1), lysoPE (18:1, 18:2, 17:1, 15:0, 16:0, 16:1, 14:0, 15:1), and linoleic acid significantly increased on the first day, while the free fatty acid content in the control group was higher than that in the treatment group on the fifth and seventh days (Figure 5b).

**Figure 6:**
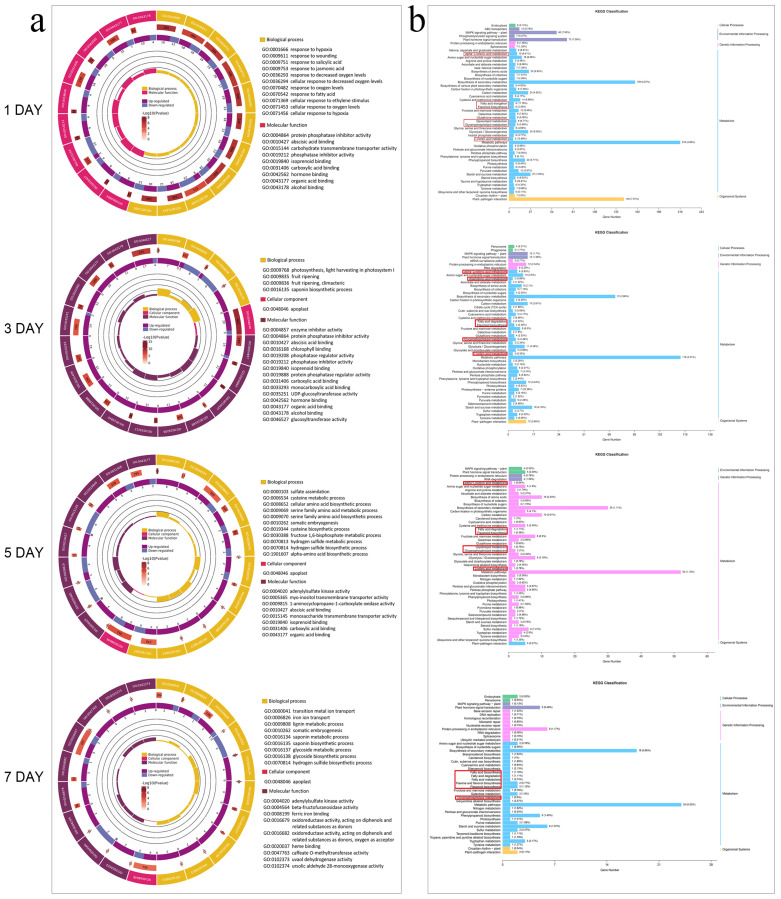
Gene Ontology (GO) and Kyoto Encyclopedia of Genes and Genomes (KEGG) annotation analysis of differentially expressed genes in the pear fruit flesh after treatment with peppermint extract. (**a**) GO analysis of differentially expressed genes at different treatment days. (**b**) KEGG analysis of differentially expressed genes at different treatment days. The contents within the red box are significant enrichment pathways related to softening.

The changes of gene expression levels in the pear fruit after treatment with peppermint extract were further analysed using GO and KEGG enrichment analyses. GO enrichment showed that the DEGs identified on the first day after treatment were mainly concentrated in pathways such as response to oxygen levels and response to hormones, whereas the DEGs on the third day were concentrated in fruit ripening. On the fifth day, genes related to ethylene synthesis were significantly enriched in the MH group (Figure 6a). The KEGG pathway analysis showed that on the first day after treatment, the alpha-linolenic acid metabolism, fatty acid elongation, flavonoid biosynthesis, glycerolipid metabolism, glycerophospholipid metabolism, and linoleic acid metabolism pathways were enriched, and there was also differential enrichment in fatty acid degradation on the third, fifth, and seventh days (Figure 6b).

**Figure 7:**
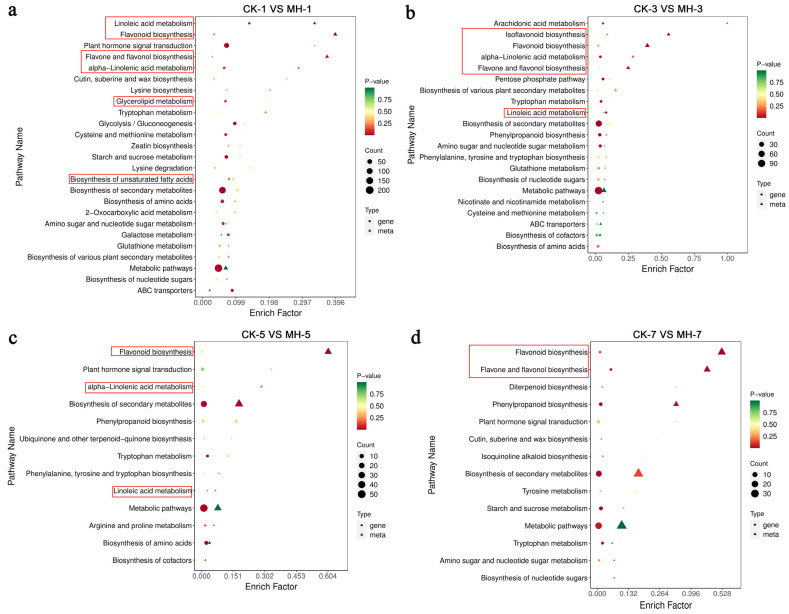
Combined metabolome and transcriptome analysis for Kyoto Encyclopedia of Genes and Genomes (KEGG) enrichment of the effect of peppermint extract (MH) treatment on fruit flesh properties on the (**a**) first, (**b**) third, (**c**) fifth, and (**d**) seventh days after treatment. The contents within the red box are significant enrichment pathways related to softening.

The combined analysis of transcriptome and metabolome data showed significant enrichment in linoleic acid metabolism and alpha-linolenic acid metabolism on the first, third, and fifth days after peppermint extract treatment (Figure 7a–c). On the first day after treatment, there was a significant difference in the biosynthesis of unsaturated fatty acids and glycerolipid metabolism (Figure 7a). Flavonoid biosynthesis consistently showed differential enrichment after treatment, with significant enrichment in both the transcriptome and metabolome data (Figure 7). In addition to the above pathways, there were significantly more pathways with common differential enrichment in the early stages after treatment than on the fifth and seventh days (Figure 7).

**Figure 8:**
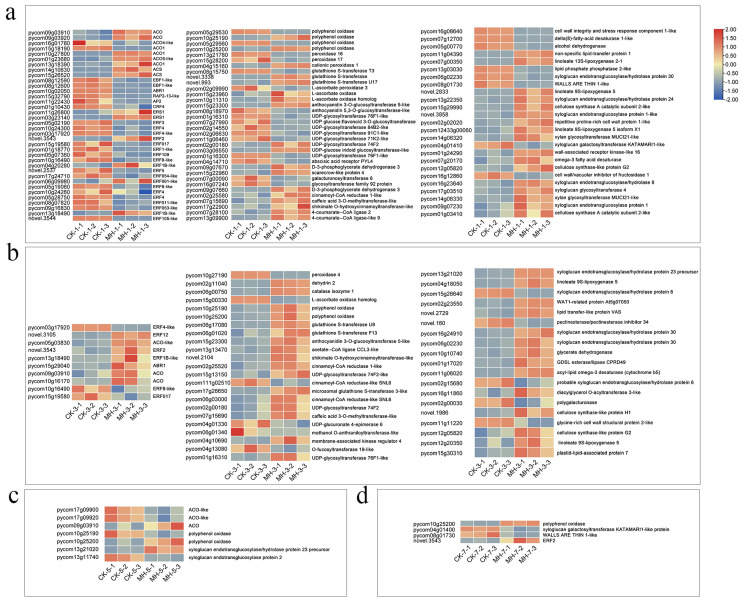
Screening of differentially expressed genes related to softening in pear flesh after peppermint extract (MH) treatment on the (**a**) first, (**b**) third, (**c**) fifth, and (**d**) seventh days after treatment. CK-1, CK-2, and CK-3 represent three repeats in the control group; MH-1, MH-2, and MH-3 represent three repeats in the treatment group.

To gain a deeper understanding of the DEGs, they were screened at different days after treatment. The Venn diagram showed a significant decrease in DEGs with increasing storage days, with 1577 DEGs identified on the first day after treatment, followed by 525, 190, and 139 DEGs identified on the third, fifth, and seventh days after treatment, respectively (Figure S1). On the first day after treatment, 36 DEGs related to ethylene were screened, among which genes involved in the ethylene signalling pathway were significantly downregulated (Figure 8a). On the third day, the number of DEGs related to ethylene decreased to 10, with most of them being upregulated (Figure 8b). On the first day after treatment, genes related to reactive oxygen species, such as PPO, POD, APX, CAT, and glutathione S-transferase (GST), were differentially expressed, and genes related to flavonoid metabolism pathways were also differentially expressed (Figure 8a). The cell wall is closely related to fruit softening. In the first 3 d after treatment with peppermint extract, the expression of genes related to lipoxygenase, xyloglucan endotransglucosylase/hydrolase 8, xyloglucan glycosyltransferase 4, xylan glycosyltransferase MUCI21-like, and xyloglucan endotransglucosylase protein 1 were significantly upregulated, indicating that fruit softening was affected by the peppermint extract in the early stage after treatment (Figure 8a,b). On the fifth and seventh days, the number of DEGs related to fruit softening was significantly reduced (Figure 8c,d).

## 4. Discussion

The storage and preservation of fruit are important postharvest processes, and the loss of fruit caused by improper preservation accounts for a considerable proportion of production losses, greatly affecting the development of the fruit industry. Therefore, researchers have been focusing on developing new preservatives to delay fruit senescence and increase economic value. As important pear varieties, European pears need to undergo postharvest ripening to reach their optimal edible state. The flesh is soluble and the juice is rich; however, the fruit is prone to spoilage after softening. Therefore, the postharvest storage process of European pears is very important for their industrial development [30]. Given their specific requirements, delaying fruit senescence cannot be achieved by inhibiting respiratory climacteric changes, which limits the ability to appropriately soften the flesh to achieve the optimal edible state. Mint has a strong bactericidal effect and is often used as a biological agent in the postharvest stage of fruit; however, its role in the storage process of European pears has not been reported to date.

In this study, we applied a simple method of soaking after boiling compared with the complex steps involved in the extraction and application of peppermint essential oil or in preparing a peppermint coating. Through soaking in mint extract, we found that the postharvest ripening and softening process of the ‘Packham’s Triumph’ pear could be delayed. On the seventh day of storage, the fruit flesh in the control group had completely dissolved and appeared to be in a watery state. By the eleventh day, the hardness had almost decreased to 0. However, the fruit treated with peppermint extract showed watery spots until the eleventh day, and the fruit still had a certain hardness that remained within the edible range. This indicated that mint extract could slow down the rate of decrease in fruit hardness and thereby prolong the optimal consumption period of ‘Packham’s Triumph’ pears. This conclusion was consistent with previous studies on the effect of peppermint in cherry tomatoes and papaya [11,15]. 

‘Packham’s Triumph’ is a typical respiratory climacteric fruit, and ethylene is the main hormone affecting fruit ripening during the postharvest process. In production, European pears could be stored for a long time in a refrigerated environment and do not undergo significant changes. However, before consumption, it is necessary to place them at room temperature to promote the respiratory climacteric, soften the flesh, and achieve the optimal edible taste. Therefore, we monitored the changes of ethylene and respiratory rate in fruit at 1–11 d after treatment. The peak values of ethylene and respiratory rate in fruit were reduced to a certain extent after peppermint extract treatment, but the extract did not delay the occurrence of the respiratory climacteric peak. Transcriptomic data showed that ethylene-related genes were significantly upregulated on the first day after treatment. Although ethylene synthesis-related genes *PcACO* and *PcACS* showed an upregulation trend, genes involved in the downstream signal transduction pathways of ethylene, *PcEIN3* and *PcERF*, were significantly inhibited by peppermint extract treatment. This indicated that peppermint extract mainly inhibited ethylene through ethylene signal transduction. As the storage time increased, the difference in ethylene-related gene expression decreased, indicating that mint extract played a role in the early stage of storage, and its effect weakened in the later stage of storage.

Previous studies demonstrated that peppermint extract contains a large amount of antioxidants, flavonoids, and polyphenols, which can enhance the antioxidant capacity of fruit [31]. Therefore, we measured the activities of SOD, POD, CAT, and APX in the fruit after soaking with peppermint extract. Indeed, we found that the mint extract could improve the antioxidant properties of fruit, with a significant increase in SOD and POD activities detected on the fifth day. This may be related to the respiratory climacteric of fruit on the fifth day, which entered the senescence process and led to the burst of reactive oxygen species. On the first day after treatment, the activities of CAT and APX were significantly higher than those of the control group, indicating that peppermint can directly induce fruit antioxidant enzyme activity and improve fruit antioxidant capacity. Transcriptomic analysis revealed differential expression of the antioxidant-related genes *PcPPO*, *PcPOD*, *PcCAT*, *PcAPX3*, and *PcGST* on the first day after treatment. Similar to the differential expression of genes involved in the ethylene signalling pathway, the expression levels of genes in the antioxidant system decreased during subsequent storage, which also confirmed that peppermint mainly played a role in the early stage after treatment. 

Many studies have shown that flavonoids have the function of enhancing antioxidant capacity [32,33]. We found that peppermint extract can significantly affect flavonoid metabolism, with a significant increase in antioxidant substances such as 7,3′,4′-trihydroxyquercetin, 3-O-methylquercetin, and kaempferol detected on the first day after treatment. Moreover, in the joint analysis of metabolomics and transcriptomics, KEGG analysis showed that flavonoid biosynthesis was differentially enriched in both datasets after peppermint treatment up to the later stage of storage. This indicated that peppermint extract could promote flavonoid metabolism, increase the accumulation of flavonoid metabolites, and enhance the antioxidant activity of fruit by regulating gene expression within the fruit. 

The softening of fruit represents a process of degradation of the fruit cell walls, in which PE, β-GAL, and α-L-Af play important roles [34,35,36]. After peppermint extract treatment, the activities of these enzymes related to cell wall degradation decreased, and the corresponding gene expression was downregulated. This indicated that peppermint extract could inhibit the softening process of fruit. Lipid metabolism is closely related to fruit softening [37]. Consistently, we found that fatty acid metabolism showed significant differential enrichment in the transcriptome and metabolome joint analysis, indicating that there were differences in the degree of fruit softening between the treatment and control groups. The results of the fruit hardness assessment also showed that peppermint extract could significantly slow down the rate of decline in fruit hardness. 

To identify the specific response process more accurately, we further screened the DEGs in the transcriptome. Among them, the genes related to xyloglucan endotransglucosylase/hydrolase (XTH) and xyloglucan glycosyltransferase were differentially expressed on the first and third days after treatment. XTH is an important class of glycoside hydrolases, playing a key role in promoting the extension of plant cell walls and in the response to stress [38]. Xyloglucan glycosyltransferase plays an important role in the synthesis of plant cell walls, having a critical catalytic role in the synthesis of xyloglucan by catalysing substrate transfer reactions [39]. The high expression of these genes indicated that peppermint extract enhanced fruit firmness by increasing the extension of plant cell walls. 

## 5. Conclusions

**Figure 9:**
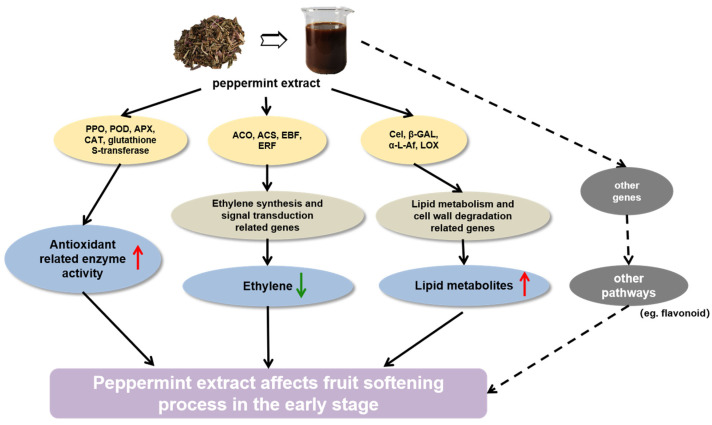
Model depicting the mechanism by which mint (*Mentha haplocalyx*) extract delays the postharvest ripening of the ‘Packham’s Triumph’ pear. The red arrow means up-regulation, and the green arrow means down-regulation.

In summary, we found that peppermint extract mainly affected the pear fruit softening process in the early stage after treatment, primarily by inhibiting the ethylene signal transduction pathway, while improving the antioxidant system of the fruit and the metabolism of related antioxidant substances such as flavonoids. During this process, the pathways related to cell wall and lipid metabolism were regulated, ultimately affecting the fruit softening process (Figure 9). In practical applications, peppermint extract had no effect on the respiratory climacteric time and maintained the normal softening process of European pears but could delay the time the fruit reached the softening state, which has great application potential.

## Data Availability

The original contributions presented in the study are included in the article/supplementary material; further inquiries can be directed to the corresponding author.

## Supplementary Materials

The following supporting information can be downloaded at https://www.mdpi.com/article/10.3390/foods13050657/s1: Figure S1: Venn diagram of the number of differentially expressed genes in the control group and treatment group on the first (blue), third (orange), fifth (green), and seventh (purple) days after peppermint extract treatment; Table S1: Information on the primers used for the gene expression analysis.
