# Supplementary material for: Mechanism of Peppermint Extract-Induced Delay of ‘Packham’s Triumph’ Pear (Pyrus communis L.) Postharvest Ripening
Source: Foods. 2024 Feb 21;13(5):657. doi: 10.3390/foods13050657 (PMC10930982; doi:10.3390/foods13050657)
Supplement: Supplementary file 1 [file foods-13-00657-s001.zip › foods-2878505-supplementary.pdf]

Supplementary data

Table S1. Information on the primers used for the gene expression analysis.

Gene name	Forward primers (5'-3')	Reverse primers (5'-3')
ACTIN	ACAGTGTCTGGATTGGAGGGTC	CATTTGGAGAACTCAGAAGCACT
PcSOD	TAGCCACGCCTAGTTCGCTA	TCCTTCTGCATTGTCTCTGCT
PcPOD	AAGGCATGCATGTGGTCAGT	CGACATATCCACCATGCCCA
PcCAT	TCAGTAAGACCGGGAAGGCA	CTTGGTAGCGTGGCTGTGAT
PcAPX	CGGCTCCGAAAGTCCTAGTT	ACGAGAGTCTTCATGTCTTGGAC
PcCel	GAGCATGTACGGCAGGGACC	TCATGGTTTCCAGGGTGCC
PcPE	ATGTTGCAGCGAAGAGGAGT	ACCTCAACCCATCACCAAGTAG
PcPG	ATCTCGTTTTGGACGTGCTC	ACGACAGCTTTCCTATTTAGTCCAT
Pcβ-GAL	TTTGGAGGCGGGCAGTATG	AACTTGCGAGCTTTGCGAAC
Pcα-L-Af	AGTCGGCTGGTGAGGAAATG	CAAGAGAGAAGCCGCTCCAT
PcLOX	AGCGACAAGAAAGAAGAACC	TACTGACCGTAGTTGATTGC

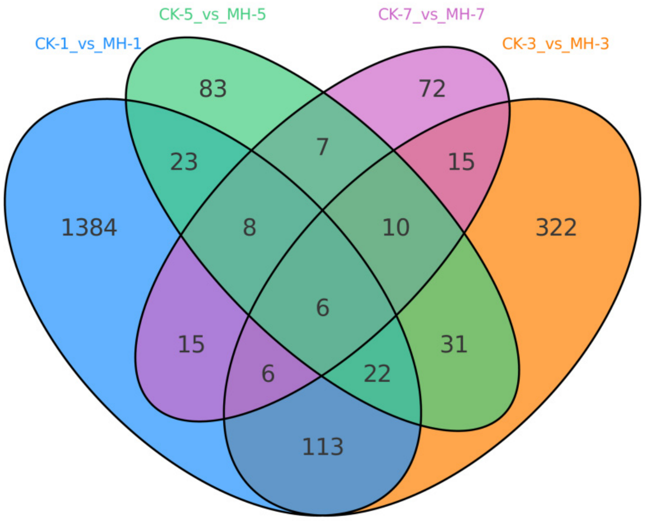

Figure S1. Venn diagram of the number of differentially expressed genes in the control group and treatment group on the first (blue), third (orange), fifth (green), and seventh (purple) day after peppermint extract treatment.
